# Building capacity in health facility management: guiding principles for skills transfer in Liberia

**DOI:** 10.1186/1478-4491-8-5

**Published:** 2010-03-18

**Authors:** Laura A Rowe, Sister Barbara Brillant, Emily Cleveland, Bernice T Dahn, Shoba Ramanadhan, Mae Podesta, Elizabeth H Bradley

**Affiliations:** 1 Yale School of Public Health, New Haven, CT, USA; 2 Mother Patern College of Health Sciences, Monrovia, Liberia; 3 Clinton Foundation HIV/AIDS Initiative, Monrovia, Liberia; 4 Ministry of Health and Social Welfare, Liberia

## Abstract

**Background:**

Management training is fundamental to developing human resources for health. Particularly as Liberia revives its health delivery system, facility and county health team managers are central to progress. Nevertheless, such management skills are rarely prioritized in health training, and sustained capacity building in this area is limited. We describe a health management delivery program in which a north and south institution collaborated to integrate classroom and field-based training in health management and to transfer the capacity for sustained management development in Liberia.

**Methods:**

We developed and implemented a 6-month training program in health management skills (i.e. strategic problem solving, financial management, human resource management and leadership) delivered by Yale University and Mother Patern College from Liberia, with support from the Clinton HIV/AIDS Initiative. Over three 6-month cycles, responsibility for course instruction was transferred from the north institution to the south institution. A self-administered survey was conducted of all participants completing the course to measure changes in self-rated management skills, the degree to which the course was helpful and met its stated objectives, and faculty members' responsiveness to participant needs as the transfer process occurred.

**Results:**

Respondents (n = 93, response rate 95.9%) reported substantial improvement in self-reported management skills, and rated the helpfulness of the course and the degree to which the course met its objectives highly. Levels of improvement and course ratings were similar over the three cohorts as the course was transferred to the south institution. We suggest a framework of five elements for implementing successful management training programs that can be transferred and sustained in resource-limited settings, including: 1) use a short-course format focusing on four key skill areas with practical tools; 2) include didactic training, on-site projects, and on-site mentoring; 3) collaborate with an in-country academic institution, willing and able to scale-up and maintain the training; 4) provide training for the in-country academic faculty; and 5) secure Ministry-level support to ensure participation.

**Conclusion:**

Our findings demonstrate key elements for scaling up and replicating educational initiatives that address management skills essential for long-term health systems strengthening in resource-poor settings.

## Background

Strengthening health systems, particularly health care delivery systems, is an international priority, as illustrated by extensive efforts to support and develop system improvements by the World Health Organization (WHO), the World Bank, major bilateral donors such as the Department for International Development (DFID) in the United Kingdom, and large private donors. Although much attention has been given to enhancing clinical and public health skills, less focus has been directed at developing management and leadership skills needed to strengthen health systems [1-4]. Adequate attention to such foundational skills is critical in order to enable large-scale, sustainable change in health care delivery in resource-limited settings.

Management skills have had a positive impact on health systems strengthening and process-related outcomes in a number of settings, including projects in Ethiopia, the Gambia, Ghana, Mozambique, Nicaragua and the United Republic of Tanzania [1-3,5-8]. These projects have demonstrated the importance of equipping managers with specific skills in priority-setting, problem-solving, and change management, and have demonstrated improvements in supervision, teamwork, planning and coordination [1-3,5,6], delivery of essential health services [3,6], and management of health resources [5,7,8]. Despite the consistency of these findings, however, evidence on the long-term sustainability and the institutionalization of such management tools is limited.

In this paper, we describe a health management capacity building program, which sought to: a) develop key management skills for County Health Teams (CHT) and health facility managers throughout Liberia, and b) transfer the capacity for sustained health management training to the country level. We examine a set of guiding principles that promotes the systematic transfer of management skills to both course participants and faculty members to ensure scalability and sustainability of the transferred skills.

Launched in 2007 at the request of the Mother Patern College of Health Sciences in Monrovia and endorsed by the Liberian Ministry of Heath and Social Welfare (MoHSW), the program was a unique partnership between the Clinton Foundation HIV/AIDS Initiative (CHAI), the Mother Patern College of Health Sciences/Stella Maris Polytechnic, and the Yale School of Public Health to strengthen management skills of key leaders in health facilities and county health teams in Liberia. We used the experience of this program to propose a set of key elements needed for programs to scale up, replicate and sustain training initiatives that enhance foundational management skills critical to providing long-term strengthening of health systems in low-income countries.

## Methods

### Setting

In 2007, after 14 years of civil war, Liberia began the process of rebuilding its health delivery system through a decentralized health care sector. As part of the rebuilding, the MoHSW developed a Basic Package of Health Services (BPHS) guaranteed to all Liberians accessing services through the public sector. Through the BPHS, health facility standards were refined and introduced at each level of care, catapulting Liberia into an environment of health facility reform. In order to meet these standards, the MoHSW focused on enhancing the management capacity of health centers, clinics, and hospitals throughout the country. In addition to these higher standards, County Health Teams (CHTs) - consisting of district health workers often with limited background or experience in management - became responsible for decision-making and priority-setting at local levels.

During this time of rebuilding and decentralization, Liberia was and is transitioning from a period of relief to one of development. With this transition have come changes in partner and donor support and an adjustment from crisis management to strategic thinking and systems development. Together, these contextual issues magnify the need for strong management skills at the CHT and health care organizational levels.

### Intervention

Within this setting of decentralization and health sector reform, a classroom-based Health Systems Management Course for health facility and CHT managers was developed and taught by Yale University, Mother Patern College, and CHAI. Follow-up and mentoring for course participants was provided by Mother Patern faculty, on-site Yale-Clinton Foundation Fellows, and CHAI staff who assisted participants in managing projects and in reinforcing course concepts. The Health Systems Management Course is a competency-based training course focused on the core skills of health care management, including scientific problem solving and strategic thinking, human resource management, financial management, and leadership development. The content of the course was designed to teach participants how to think strategically in several key domains and then how to use the more conceptual tools to solve concrete problems, including the implementation of the Basic Package of Health Services and related policies. A core component of the training was to link classroom didactics to field-based applications, where teams were expected to use the management skills developed and apply them to specific expectations for MoHSW policy implementation. For example, problem statements were generated around expected deliverables outlined in the six focus areas of the Basic Package of Health Services (e.g. maternal and infant health, child health, reproductive and adolescent health, communicative disease control, mental health and emergency care) such as establishing regular supportive supervision in primary health care units, ensuring effective management of malaria, and establishing an effective county referral system.

Course participants included two to four managerial representatives from CHTs, the MoHSW, government hospitals, and Liberians working with international non-governmental organizations (INGOs) in the oversight of multiple health facilities. Participants were nominated to apply and selected based on successful completion of a course application, which included holding a supervisory or management position within a CHT, government institution, INGO or faith based organization; answering four short questions in a clear manner; and coming recommended by the head of one's organization or CHT.

Consistent with the goal of building capacity at Mother Patern College and transitioning the course from Yale to Liberian faculty, the course was conducted in a stepped approach with increasing responsibility transferred to Mother Patern instructors with each training cycle. Each cycle comprised three classroom periods lasting approximately 10 days each, which took place over a period of five months. In the first cycle, Yale University instructors developed and taught 100% of the material. In the second cycle, after substantial train-the-trainer sessions, instructors from Mother Patern College conducted 50% of the training with Yale facilitators conducting the remaining 50% in addition to providing supervision and feedback. In the third cycle, Mother Patern College took on 100% of course instruction, with Yale providing only back-up support. By the fourth cycle, Mother Patern instructors will teach and manage 100% of the material without Yale or CHAI support. The full transfer (the first three cycles or cohorts where Yale and CHAI were present) required approximately 2 years to complete.

In addition to classroom training, the course required substantial field-based work with supervision. Between the 10-day classroom periods, participants returned to their counties and/or health facilities to apply course skills and tools in their work settings, and to complete field-based project assignments including the use of root cause analysis balanced scorecards and comparative analysis in order to support efficient problem solving approaches. During this time, participants received on-the-ground mentoring in management skills and quality improvement approaches, as they applied classroom learning to their real-world work situations. Mentoring was provided by Mother Patern faculty, CHAI, the MoHSW, and five Yale-CHAI Fellows placed in two Monrovia hospitals and on a rotational basis in CHTs throughout Liberia.

Since the completion of the program, staff originally trained through the train-the-trainer mechanism have identified and trained additional individuals. The original cohorts of trainees are now conducting trainings themselves targeting middle-level staff in counties including clinical officers-in-charge, supervisors and coordinators. The original management course material has been used to prepare these subsequent trainings. In the 6 months following the final cohort reported in this paper, graduates of the original program have trained 27 additional staff from two counties, with plans to expand to three more counties in the next 6 months. In addition, the Liberian academic partner, Mother Patern College, is currently working from the original materials to mentor the MoHSW in developing its own management and leadership program. Through interviews and an on-the-ground assessment, Mother Patern College management staff members are conducting a program evaluation in order to measure the course's impact and identify areas for improvement, as needs and content continually evolve in the Liberian context. These efforts reflect the transfer of the program beyond the first generation of trainees and underscore the degree to which both country-level and Ministry-level staff continue to integrate the material into their positions.

### Data collection and measures

We conducted a self-administered survey of all participants completing the course at the end of each session to measure change in key indicators as the transfer process occurred (cohort 1 was taught 100% by Yale, cohort 2 was taught 50% by Yale and 50% by Mother Patern College, and cohort 3 was taught 100% by Mother Patern College). Indicators included self-rated management skills (before and after the course), course evaluations, and faculty members' responsiveness to participant needs and ability to teach and manage the training. Participants rated each indicator on a scale, with possible responses tailored to the survey item. For respondents' self-rated management skills, response categories were: very strong and confident, strong, moderate, weak, and very weak. For course evaluations, the response categories were: extremely well, well, a little, not at all, no opinion. For faculty members' ability to teach and manage the course effectively, response categories were: yes, definitely; yes, somewhat; no; not at all; and no opinion. Evaluations were designed by the research team and took approximately 30 minutes to complete (see Additional File 1).

### Data analysis

We utilized data from surveys administered in the final session for each cohort. We used standard descriptive statistics to characterize item responses, stratified by cohort. We evaluated differences between cohorts for the items of interest using Fisher's exact tests. We calculated 95% confidence intervals around proportions using Wilson's method, including a continuity correction [9]. Analyses were conducted using SAS version 9.1 (SAS Institute, SAS. 2003: Cary, NC.).

## Results

### Study participants

Between June 2007 and January 2009, we trained 97 participants, representing all 15 counties in Liberia, the National AIDS Control Program, two hospitals, the MoHSW and four INGOs. A total of 93 participants completed surveys, yielding a response rate of 95.9%. The first training cohort (June 2007 - October 2007) consisted of 36 participants representing 7 counties and the National AIDS Control Program. The second training cohort (January 2008 - May 2008) consisted of 32 participants from the remaining 8 counties and 1 government hospital. The third training cohort (October 2008 - January 2009) consisted of 28 participants including additional members of 4 CHTs, 2 hospitals, the MoHSW and 4 INGOs. Each county and/or facility was represented by 2-4 members of their management team, including County Health Officers, County Health District Administrators, Community Health Department Directors, County Health Services Administrators, Nursing Directors and Hospital Administrators.

### Respondents' views

In the area of self-assessed personal management skill development, significantly higher proportions of respondents rated their management skills upon completing the course as "strong" or "very strong" in comparison to the beginning of the course in all three cohorts (P-value < 0.001). In general, at least two-thirds of the respondents indicated the course met each objective "extremely well" (Table 1). In the area of faculty responsiveness, most respondents reported that faculty "definitely" responded effectively to questions and "definitely" related theory to real-life by using workplace problems. Finally, nearly all respondents reported they would "definitely" recommend the course to colleagues.

**Table 1:** Comparison of participant perceptions of course offerings across three cohorts (per cent positive responses presented with 95% confidence intervals [CIs])

Evaluation Question	Cohort 1 (Yale) n = 35% (95% CI)	Cohort 2 (Yale/M. Patern) n = 32% (95% CI)	Cohort 3 (M. Patern) n = 26% (95% C
**Self-reported management skills (% reporting "very strong and confident")**			
**Self-rated management skills:**			
Before the course	17.7 (7-34)	15.6 (6-34)	3.9 (0-22)
After the course	100.0 (87-100)	96.9 (82-100)	100.0 (83-100)
**Course ratings (% reporting "extremely well")**			
**How well did the training:**			
Address new management techniques?	91.4 (76-98)	96.9 (82-100)	88.5 (69-97)
Build management capacity in the areas of leadership, strategic planning, human resource development, and financial management?	67.7 (49-82)	78.1 (60-90)	76.9 (56-90)
Create a community of learners and build teams of healthcare professionals?	57.1 (40-73)	80.0 (61-92)	69.2 (48-85)
**How well did the training meet its objectives for:**			
Scientific method of problem solving and related skills/tools?	80.0 (63-91)	96.8 (81-100)	84.6 (64-95)
Personal and professional leadership?	67.7 (49-82)	86.7 (68-96)	70.8 (49-87)
Building teams, personal communication styles, and empowering employees	78.1 (60-90)	87.1 (69-96)	69.2 (48-85)
**Faculty's ability to teach and manage the training (% reporting "yes, definitely")**			
Faculty responded effectively to questions	88.6 (72-96)	96.9 (82-100)	88.5 (69-97)
Faculty related theory to real-life by using actual workplace problems or concerns in their teaching	88.2 (72-96)	100.0 (87-100)	92.3 (73-99)
If the course were offered again, would you recommend it to your colleagues?	93.9 (78-99)	100.0 (85-100)	100.0 (83-100)

*Note: Differences across cohorts were non-significant (P-values > 0.05) for all items using Fisher's exact test

All cohorts comprised the same level of employees; however, the individuals in the third cohort had modestly lower self-rated management skills than those in the first two cohorts. This may be due to the inclusion of some less experienced staff in the later cohort and greater recognition in Liberia of what management skills included as the Basic Package of Health Services was implemented; however, the differences in baseline self-rated management skills of the third cohort was not statistically significant (P-values > 0.05).

### Differences in participants' views across cohorts

As a major goal of the course was to transition responsibility from Yale to Liberian faculty, we examined differences in participants views and satisfaction between the first cohort (taught completely by Yale faculty), the second cohort (taught with faculty time split evenly between Yale and Mother Patern College) and the third cohort (taught completely by Mother Patern College). There was no significant difference in participants' rating of the course in any areas (all P-values > 0.10), suggesting that the transition from Yale to Liberian faculty was effective.

## Conclusions

Effectively transferring programs is a central challenge for partnerships involving donor and host institutions. In this study, we illustrate the development and successful transition of a management course from a northern to southern institution including relevant alterations to course content and new methods of delivering material to participants. None of the response patterns differed significantly across the three cohorts, which we believe suggests the presence of a strong and effective collaboration between the partnering institutions as the course was transferred to Mother Patern College. The successful transfer of the management training to the College, through the train-the-trainer mechanism and through the College's and instructor's vision and dedication, demonstrates the program's impact in terms of effectively incorporating and enabling the College to offer subsequent management courses.

Based on our experience through this health management program, a set of five guiding principles for developing management skills and local capacity for instruction has emerged. These five principles can assist programs in other countries, particularly in post-conflict settings, attempting to implement similar initiatives that address transferring, scaling up and sustaining management and leadership skills.

### 1. Use a short-course format focusing on key skill areas with practical tools that are specific and replicable

Using a short-course format (3 sessions, 1-2 weeks per session) minimized work-place disruption, while allowing for focused didactic training and group work sessions on practical management components and specific areas of need. Such a format has proven effective in addressing health systems strengthening in a variety of other settings including a project to improve district-level health team management in The Gambia [1], a public health management development project in Nicaragua [2], and a development program for primary health care workers in Mozambique [6]. The course focused on four core management skills identified as critical areas for strengthening health systems [10,11], providing participants with a framework for essential management systems while allowing for effective organization of course material.

### 2. Integrate classroom training and field-based, mentored projects

The integration of classroom, didactic training and field-based, mentored projects was key to bringing together classroom-based training with practical problems and real-life relevance. An important part of the training required participants to complete between-session "assignments," or implementation plans, grounded in specific problems from participants' work environments and formally presented at course completion. As management skills are most effectively learned through methods of trial and error rather than through classroom lectures alone, the 'learning by doing' approach [1,11,12] between sessions allowed participants to practice management tools in their own work setting, with the participation of colleagues and support of on-site mentors. Mentoring, in combination with classroom training, has been found to improve performance and support the effective application of new skills within one's work environment [2,3,13-15]. This combined approach allowed participants to bring obstacles faced and issues addressed back to subsequent sessions for input and discussion.

### 3. Collaborate with an in-country institution willing and able to take full responsibility for scaling-up and maintaining the training

Without a mechanism to ensure course continuation once partnering organizations have left, dissemination of the course's management skills would be limited to a handful of individuals chosen during a small window of time. One of the biggest challenges of management development is ensuring scalability and replication in-country [12]. By conducting the training in a stepped approach, in collaboration with an in-country sponsoring institution with the ability and willingness to administer, instruct and ultimately absorb the course into their own curriculum, and with the ability to adapt the program to meet the changing needs of health care professionals over time, a continual cadre of health care managers can be trained throughout Liberia. Such collaborative approaches increase opportunities to build long-term, sustainable change [2,11,16,17].

### 4. Provide train-the-trainer sessions for in-country institution faculty who will teach the training in the future

As each successive training round entailed increased hand-over of teaching and facilitation responsibility to Mother Patern faculty, training and mentoring faculty through course instruction was imperative. This step was vital to the transfer process and for long-term sustainability of the training as in-country faculty are now available to meet the continual management needs of Liberia's health care professionals [2]. Additionally, the train-the-trainer sessions allowed Mother Patern faculty to provide invaluable contextual insight into specific examples, case studies, and role plays, while allowing them to gain ownership and familiarity over the material. There was limited turnover in Yale faculty, CHAI staff, or Mother Patern faculty during the process, which helped ensure continuity in learning and transferring skills. The curriculum is now fully documented and a core group of Liberian faculty has the ability to train others, as staff may transition in the future.

### 5. Secure Ministry of Health support in order to enhance county and facility participation

The endorsement and support of the course by Liberia's MoHSW was and is a central component to the program's implementation and its continuation at Mother Patern. The program's management content was fully embraced by the MoHSW as they became a driving force behind its implementation. Additionally, collaboration and input from MoHSW staff in the areas of human resources and financial management was essential to tailor topics to specific Ministry policies being established as the trainings were conducted. Strong support from senior administration within a country is critical to facilitating change [1,3,7,12,18]. Such support re-emphasizes the importance and value of program content for participants and provides a foundation upon which scalability and sustainability are possible.

Several limitations should be considered when interpreting our findings. First, this was an exploratory study, with a modest sample size. Although we had a high response rate (96%), small differences between cohorts could not be detected with our limited statistical power. Second, substantial environmental changes took place over the course of the program. For instance, the BPHS implementation was at a more advanced stage by the time of the final cohort's training. This may have affected the course ratings, although it is difficult to anticipate the direction of that effect. Third, although in all three cohorts participants self-reported management skills improved dramatically over the duration of the course, we did not have objective measures of facility-level management improvements or outcomes that could be related to these self-reports, limiting our ability to evaluate the full impact of the course. Fourth, the study did not control for social response bias, in which respondents respond positively to please the program staff. This may have occurred due to immediate post-course administration of surveys in the physical location of trainers; however, surveys were coded so that responses would be anonymous and staff members who were not the trainers conducted the surveys. Additional qualitative data may have provided added insight to understanding participants' experiences in the course, but we did not have resources to conduct such analyses. Fifth, we were unable to examine whether subsets of the design elements would have been effective without all aspects. Nevertheless, we believe, based on the experience, that additional attention to each of the five principles is most likely to be effective in transferring the program to the in-country partner. Sixth, we do not have quantitative data on the impact of the program or long-term evidence about its continuous offering. However, as described in the results, the program and second-generation versions of the program are still in operation in Liberia. Finally, the study took place in one country with an academic partner that was able to absorb and integrate the training into their curriculum. Our experience may have differed in other settings without this kind of institutional support. As a result, we believe more research is needed in this area to replicate and extend the conclusions drawn from these early results in Liberia.

Strong leadership and effective management are critical skills needed to direct large-scale sustainable change. This is particularly true during health sector reform or decentralization as responsibilities and decision-making are often shifted onto individuals without prior management experience [1,2,6,12]. By focusing on core management and leadership concepts and by working to institutionalize the concepts in a local capacity, this program addresses the central health sector development challenges of sustaining and replicating initiatives in order to improve healthcare delivery. A successful North-South collaboration, grounded in the five principles outlined in the transfer framework above, can ensure transferability and program sustainability, while enabling improvements within a delivery system even as the system changes over time.

## Competing interests

The authors declare that they have no competing interests.

## Authors' contributions

All authors collaboratively conceived of this article. LAR had primary responsibility for the original manuscript draft with substantial co-writing and intellectual content being contributed by SBB, EC, SR, MP and EHB. All authors have approved the final draft.

## Supplementary Material

Additional file 1 Health Systems Management Course, Mother Patern College of Health Sciences: Course evaluation - Cohort X Session X. Click here for file
