# Supplementary material for: Building capacity in health facility management: guiding principles for skills transfer in Liberia
Source: Hum Resour Health. 2010 Mar 18;8:5. doi: 10.1186/1478-4491-8-5 (PMC2850875; doi:10.1186/1478-4491-8-5)
Supplement: Additional file 1 — Health Systems Management Course, Mother Patern College of Health Sciences: Course evaluation - Cohort X Session X. [file 1478-4491-8-5-S1.DOC]

**Health Systems Management Course**

**Mother Patern College of Health Sciences**

**Course evaluation – Cohort X Session X**

Please complete the following evaluation to assist us in improving the content and design of this course in the future.

| **1) What is your primary role?**  **(Please check one box)** | County Health Officer  County Health Services Administrator  Nursing Director  Ministry of Health and Social Welfare Representative  (please specify ___________________________________________________)  Other ___________________________________________________________ | | | | | | | |
| --- | --- | --- | --- | --- | --- | --- | --- | --- |
| **2) How would you describe your management skills before you started the course?** | Very strong and confident | Strong | | Moderate | | Weak | | Very  weak |
| **3) How would you describe your management skills at this point in the course?** | Very strong and confident | Strong | | Moderate | | Weak | | Very  weak |
| **4) Overall, how helpful did you find the training in addressing new management techniques?** | Extremely  helpful | Helpful | | Not very  helpful | | I learned  nothing new | | No  opinion |
| **5) Overall, how well did the training meet its specific objectives as stated in the syllabus?** | | | | | | | | |
| **a. To foster a more widespread understanding of the vision of the National Health Plan and the leadership and management skills required for implementation.** | Extremely  Well | Well | | A Little | | Not At All | | No  opinion |
| **b. To build management capacity in the areas of leadership, strategic planning, human resource development, and financial management.** | Extremely  Well | Well | | A Little | | Not At All | | No  opinion |
| **c. To create a community of learners and build teams of healthcare professionals that can work together to strengthen the Liberian health sector.** | Extremely  Well | Well | | A Little | | Not At All | | No  opinion |
|  | | | | | | | | |
| **6) Did the training meet its specific objectives for the following topics:** | | | | | | | | |
| **a. Scientific method of problem solving and related skills/tools?** | Extremely  Well | | Well | | A Little | | Not At All | No  opinion |
| **b. Project implementation, including change management?** | Extremely  Well | | Well | | A Little | | Not At All | No  opinion |
| **c. Personal and professional leadership?** | Extremely  Well | | Well | | A Little | | Not At All | No  opinion |
| **d. Building teams, personal communication styles, and empowering employees?** | Extremely  Well | | Well | | A Little | | Not At All | No  opinion |
| **7) Did practicing some of the concepts taught in the small-group exercises increase your understanding of how to use them?** | Yes, definitely | | Yes,  somewhat | | No | | Not at all | No  opinion |
| **8) Did the presenters respond effectively to questions raised?** | Yes, definitely | | Yes,  somewhat | | No | | Not at all | No  opinion |
| **9) Did the presenters relate theory to real-life by using actual workplace problems or concerns in their teaching?** | Yes, definitely | | Yes,  somewhat | | No | | Not at all | No  opinion |

**THANK YOU FOR COMPLETING THIS EVALUATION**

| **10) Did the presentation materials and handouts convey the material that was being presented?** | | Yes, definitely | | Yes,  somewhat | | No | | Not at all | | No  opinion |
| --- | --- | --- | --- | --- | --- | --- | --- | --- | --- | --- |
| **11) How well were you able to follow and understand different methods for presenting training material?** | |  | | | | | | | | |
| **a)** PowerPoint Presentations | | Extremely Well | | | Well | | A Little | | Not at All | |
| **b)** Written & Distributed Manual | | Extremely Well | | | Well | | A Little | | Not at All | |
| **12) Overall, how would you describe the pace of the course?** | | Much  too fast | A little  too fast | | Just right | | A little too slow | | Much too  slow | |
| **13) If the course were offered again, would you recommend it to your colleagues?** | | Yes, definitely | Yes,  maybe | | Not likely | | Not at all | | No  opinion | |
| **14) Overall, what was the most effective or useful exercise for you from the training? Why?** |  | | | | | | | | | |
| **15) Please comment on how you felt the facilitators taught the course. Were their particular strengths or weaknesses of the facilitators?** |  | | | | | | | | | |
| **16) How could the training have been improved?** |  | | | | | | | | | |
| **17) Please feel free to add any more comments or suggestions you believe to be useful.** |  | | | | | | | | | |
